# Associations between long-term exposure to air pollution and blood pressure and effect modifications by behavioral factors

**DOI:** 10.1016/j.envres.2019.109109

**Published:** 2020-03

**Authors:** Na Li, Gongbo Chen, Feifei Liu, Shuyuan Mao, Yisi Liu, Suyang Liu, Zongfu Mao, Yuanan Lu, Chongjian Wang, Yuming Guo, Hao Xiang, Shanshan Li

**Affiliations:** a Department of Global Health, School of Health Sciences, Wuhan University, Wuhan, China; b Global Health Institute, Wuhan University, Wuhan, China; c Department of Environmental and Occupational Health Sciences, University of Washington, 1959 NE Pacific Street, Seattle, USA; d Environmental Health Laboratory, Department of Public Health Sciences, University Hawaii at Manoa, 1960 East West Rd, Biomed Bldg, D105, Honolulu, USA; e Department of Epidemiology and Biostatistics, School of Public Health, Zhengzhou University, Zhengzhou, China; f Department of Epidemiology and Preventive Medicine, School of Public Health and Preventive Medicine, Monash University, Melbourne, Australia; g Hubei Biomass-Resource Chemistry and Environmental Biotechnology Key Laboratory, Wuhan University, Wuhan, China

**Keywords:** Air pollution, Blood pressure, Hypertension, Rural China, Effect modification

## Abstract

**Background:**

Studies on the hypertensive effect of long-term air pollution exposure were inconclusive and showed scarce evidence from rural areas in developing countries. In this context, we examined the associations of air pollution exposure with hypertension and blood pressure, and their effect modifiers in rural Chinese adults.

**Methods:**

We studied 39,259 participants from a cohort established in five rural regions of central China. Individual exposures to PM_2.5_ and PM_10_ (particulate matter with an aerodynamic diameter less than or equal to 2.5 μm and 10 μm) and nitrogen dioxide (NO_2_) was evaluated using satellite-based spatiotemporal models. Mixed-effect regression models were applied to examine the associations of long-term exposure to air pollution with hypertension and four blood pressure component measurements, including systolic blood pressure (SBP), diastolic blood pressure (DBP), mean arterial pressure (MAP) and pulse pressure (PP). Several potential effect modifiers related to demographic and behavioral factors were also examined.

**Results:**

The results showed that for each 1 μg/m^3^ increase in PM_2.5_, PM_10_ and NO_2_, the adjusted odds ratio of hypertension was 1.029 (95%CI: 1.001,1.057), 1.015 (95%CI: 1.001, 1.029) and 1.069 (95%CI: 1.038, 1.100), respectively. These three air pollutants were also associated with increased SBP (except for PM_10_), DBP and MAP. The hypertensive effects of air pollution were more pronounced among males, smokers, drinkers, individuals with a high-fat diet, and those with high-level physical activity.

**Conclusion:**

Long-term exposure to PM_2.5_, PM_10_ and NO_2_ was associated with increased blood pressure and hypertension in rural Chinese adults, and the associations were modified by several behavioral factors.

## Highlights

• The study aimed at rural population in developing countries.
• The effect estimates of NO_2_ was observed to be larger than PM_2.5_ and PM_10_.
• Pulse pressure was negatively associated with air pollution exposure.
• Several behavioral factors can modify the hypertensive effect of air pollution.

## Graphical abstract

**Figure:**
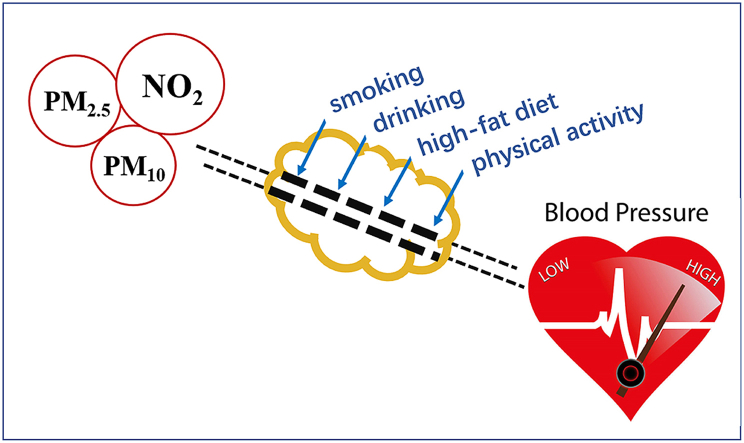

## 1 Introduction

High systolic blood pressure (SBP) was ranked as the leading risk factor for mortality and disability globally (Global Burden Disease Risk Factor Collaborators, 2018). Particularly in China, high SBP was the top risk factor for mortality and accounted for 2.54 million deaths in 2017, of which 95.7% were due to cardiovascular diseases (Zhou et al., 2019). Moreover, particulate matter pollution was the fourth leading contributor to both deaths and DALYs in China in 2017 (Zhou et al., 2019). The associations of air pollution exposure with increased cardiovascular morbidity and mortality have been revealed by a large amount of epidemiological evidence, and changes in blood pressure were proposed to be a pathway to explain the association at the functional level.

Accumulated epidemiological studies also evaluated associations of long-term exposure to air pollution with blood pressure and hypertension (Xie et al., 2018; Yang et al. 2018a, 2019a, 2019b; Zhang et al., 2019). However, most of the research were primarily concerned with urban areas and developed countries, and the results remained inconsistent. A recent review focusing on vulnerable populations for adverse cardiovascular outcomes from air pollution indicated that more researches in rural settings and developing countries were warranted, in particular because of high concentration of air pollutants and large burden of cardiovascular disease in such areas (Tibuakuu et al., 2018). Rural China is faced with worsening air pollution with the rapid urbanization and economic progress. In addition, the hypertension prevalence among rural Chines adults has shown a persistent upward trend since 1993, and disparity between urban and rural region in hypertension prevalence has gradually narrowed (Li et al., 2017). As rural residents account for more than half of the Chinese population, it could be beneficial for hypertension prevention and public health improvement to explore the long-term effects of air pollution exposure on blood pressure and prevalent hypertension among rural Chinese.

The adverse effects of air pollution were not the same for all individuals (Tibuakuu et al., 2018). It is therefore imperative to identify effect modifiers of the associations between air pollution exposure and health outcomes, and to understand the factors that increase vulnerability to the health effects of air pollution. Studies show inconsistency in examining effect modifiers in the relationships between long-term air pollution exposure and cardiovascular outcomes. For example, some studies observed higher risks for cardiovascular outcomes for smokers (Pope et al., 2009; Turner et al., 2017; Xie et al., 2018), whereas some observed higher risks for nonsmokers (Lin et al., 2017a; Pope et al., 2002; Zhang et al., 2018), and some did not find such effect modification by smoking status (Liu et al., 2017).

In this context, we investigated the associations of three-year exposure to air pollutants (PM_2.5_, PM_10_ and NO_2_) with hypertension and four blood pressure components, including SBP, diastolic blood pressure (DBP), mean arterial pressure (MAP) and pulse pressure (PP), based on the Henan Rural Cohort Study in central China.

## 2 Methods

### 2.1 Study population

The study population were from a Cohort Study carried out in Henan Province, China in 2015. This province had a relatively high density of population and severe air pollution. The study design and inclusion criteria of the Henan Rural Cohort Study have been described in our previous publications (Tian et al. 2018a, 2018b). In brief, a multi-stage stratified cluster sampling method was employed to recruit participants from the general adult residents. First, we selected one rural county from each southern, central, northern, eastern, and western region of Henan Province using a simple cluster sampling approach. They are Tongxu county, Yima county, Suiping county, Xinxiang county and Yuzhou county. Second, rural communities (referred to as a “township”) between one and three in each county were selected according to the stability of population, local medical conditions and the compliance of the residents. Third, we investigated all eligible candidates in each rural village (the administrative unit) of the selected townships. Overall, we sent out a total of 41,893 invitations to those who met the inclusion criteria. However, 2634 individuals did not response to the baseline survey, resulting 39, 259 participants finally included (response rate: 93.7%) (Liu et al., 2018). All the participants signed the informed consents before interviews and data collection.

### 2.2 Assessment of hypertension and blood pressure

Hypertension cases were defined as systolic blood pressure of 140 mmHg or higher or diastolic blood pressure of 90 mmHg or higher, or either self-reported physician-diagnosed hypertension or current intake of any antihypertensive medication, or both (Liu, 2011). We measured blood pressure for each participant using an electronic sphygmomanometer (HEM-770A Fuzzy, Omron, Kyoto, Japan), according to the American Heart Association's standardized protocol (Perloff et al., 1993). Participants were asked not to smoke, drink alcohol, have coffee or tea, and to abstain from exercising for at least 30 min before measuring blood pressure. The mean of three consecutive measurements was calculated as the blood pressure measurement of each participant. MAP was calculated by DBP +1/3 (SBP − DBP) and PP was calculated as SBP minus DBP (Darne et al., 1989).

### 2.3 Assessment of air pollution exposure

We estimated daily concentration of PM_2.5_, PM_10_ and NO_2_ at a spatial resolution of 0.1° (approximately equal to 10 km) with machine learning algorithm using ground-monitored air pollution data, satellite-retrieved aerosol optical depth (AOD) and the information on other spatial and temporal predictors (urban cover, forest cover, weather data, and calendar month, etc.). The detailed description of the estimation has been previously published (Chen et al. 2018a, 2018b). The ability of prediction was tested by a ten-fold cross-validation, and the results showed that R^2^ for daily PM_2.5_, PM_10_ and NO_2_ prediction was 83%, 78% and 64%, respectively. Each participant's daily exposure to air pollutants during the three years prior to the baseline survey was estimated according to the geocoded address of their natural village (latitude and longitude) through AutoNavi Map ([Anonymous], 2019). Then the estimated daily concentrations were aggregated into three-year average concentrations that was used in the analyses.

### 2.4 Assessment of covariates

All the variables in the current analyses were collected through face-to-face interviews by trained investigators. Sociodemographic variables included age, sex and home address. Socioeconomic variables were educational attainment, marital status and average monthly income. Educational attainment was categorized into three groups: low (illiteracy or primary school), medium (junior school), and high (senior high school or above). Behavioral variables included smoking status, alcohol drinking, high fat diet, more vegetables and fruits intake and physical activity. Smoking was defined as a person who smoked more than one cigarette per day in the past six months. Drinking was defined as consumption of alcoholic drinks for twelve or more times in the past one year, whether spirits, beer, wine, or other forms of alcohol beverage. Smoking status was grouped into “never smoked” and “ever smoked” (those who were former or current smokers). Alcohol drinking was also divided into “never drunk” and “ever drunk”. A high fat diet referred to consumption of 75 g or more meat from livestock and poultry per day, and “more vegetables and fruits intake” referred to average intake of 500 g or more vegetables and fruits per day, on recommendations of Chinese dietary guidelines (Wang et al., 2016). The level of physical activity was categorized according to the international physical activity questionnaire (IPAQ) (Lee et al., 2011). The diagnosis of type 2 diabetes was based on the American Diabetes Association recommendations (American Diabetes Association, 2013).

### 2.5 Statistical analysis

Participants with missing blood pressure measurements, hypertension status, or other key covariate data (n = 52) were excluded from the analyses, resulting a final study population of 39,207 rural Chinese adults for the current analyses.

We employed generalized linear mixed models with a random effect term for survey sites to examine associations of air pollutants with blood pressure measurements and hypertension prevalence. The effect estimates for four blood pressure component measurements were calculated from linear regression and reported as changes in mmHg for each 1 μg/m^3^ increase in three-year average concentration. For hypertension, the effect estimates were reported as odds ratios (ORs) (per 1 μg/m^3^ increase) and 95%CI. We controlled for potential confounders based on the previous literature on air pollution and blood pressure. We initiated the model development with a crude model (no adjustment), and then added a range of covariates into regression models based on previous literature. All the models were adjusted for sex, age, educational level, marital status, monthly individual income, smoking, drinking, high-fat diet, more vegetables and fruits intake, physical activity, BMI, family history of hypertension, type 2 diabetes and survey sites. Among these covariates, survey site was included as random effect term and the remaining cofounders were incorporated as fixed effect terms. This method has also been widely applied in previous studies exploring the long-term effect of air pollution on health (Lin et al., 2017b; Yang et al., 2018b; Zhang et al., 2019). In view of the high co-linearity among three air pollutants, only single-pollutant models were applied in our analysis (Spearman correlation coefficients were 0.95 for PM_2.5_ and PM_10_, 0.90 for PM_2.5_ and NO_2_, 0.96 for PM_10_ and NO_2_. See Table S1 in supplementary material). In addition, we performed several stratified analyses to test potential effect modifications of sex, age, smoking, drinking, high-fat diet, more vegetables and fruits intake and physical activity by including an interaction term. (Liu et al., 2017).

We conducted several sensitivity analyses. First, we excluded participants taking anti-hypertensive medicines to examine the associations. Second, we restricted study participants to those who were free of obesity and type 2 diabetes to reduce the inﬂuence from these factors that are potentially on the causal pathway between air pollution and hypertension.

All analyses were completed using R version 3.5.0.

## 3 Results

### 3.1 Descriptive statistics

The location of five survey sites in the Henan Rural Cohort Study was displayed in Fig. S1 in supplementary material. The basic characteristics of all participants are shown in Table 1, and characteristics of participants by study sites were shown in Table S1. We excluded 34 participants because of the missing information on blood pressure measurement or hypertension status, and a total of 39,207 participants were finally included. There were 12,823 hypertension cases identified, with the prevalence of 32.7%. Among the hypertension cases, 7879 (61.4%) were self-reported and 4955 (38.6%) were diagnosed in the baseline survey. Moreover, 6319 hypertensive participants (49.3% of the whole hypertensive participants) had taken anti-hypertensive medication during the two weeks prior to the survey.

**Table 1:** Baseline characteristics of study participants.

Characteristics^a^	Non-hypertension (n = 26,384)	Hypertension (n = 12,823)	Total (n = 39,207)	*P* value
Age, years	53.27 ± 12.43	60.39 ± 10.09	55.6 ± 12.18	<0.001
Body mass index, kg/m^3^	24.25 ± 3.36	26.04 ± 3.67	24.83 ± 3.56	<0.001
Systolic blood pressure, mmHg	115.82 ± 11.91	146.81 ± 16.86	125.95 ± 19.99	<0.001
Diastolic blood pressure, mmHg	72.58 ± 8.14	88.22 ± 10.67	77.70 ± 11.64	<0.001
Mean arterial pressure, mmHg	87.00 ± 8.64	107.75 ± 11.12	93.78 ± 13.62	<0.001
Pulse pressure, mmHg	43.23 ± 8.68	58.59 ± 14.50	48.26 ± 13.09	<0.001
3-year average exposure				
PM_2.5_, μg/m^3^	73.22 ± 2.61	73.86 ± 2.43	73.42 ± 2.57	
PM_10_, μg/m^3^	131.95 ± 5.93	133.52 ± 5.42	132.46 ± 5.81	
NO_2_, μg/m^3^	39.59 ± 3.66	40.45 ± 3.43	39.87 ± 3.60	
Sex				0.170
male	10,348 (39.2)	51,272 (39.9)	15,470 (39.5)	
female	16,036 (60.8)	7701 (60.1)	23,737 (60.5)	
Educational level				<0.001
Low	10,722 (40.6)	6826 (53.2)	17,548 (44.8)	
Medium	11,210 (42.5)	4413 (34.4)	15,623 (39.8)	
High	4452 (16.9)	1584 (12.3)	6036 (15.4)	
Marital status				<0.001
Married/Cohabitating	24,023 (91.1)	11,173 (87.1)	35,196 (89.8)	
Widowed/Single/Divorced/Separation	2361 (8.9)	1650 (12.9)	4011 (10.2)	
Income				<0.001
≤500 yuan	8924 (33.8)	5073 (39.6)	13,997 (35.7)	
500–1000 yuan	8667 (32.8)	4221 (32.9)	12,888 (32.9)	
≥1000 yuan	8793 (33.3)	3529 (27.5)	12,322 (31.4)	
Smoking				0.131
Never	19,145 (72.6)	9395 (73.2)	28,540 (72.8)	
Ever	7239 (27.4)	3428 (26.7)	10,667 (27.2)	
Drinking				0.010
Never	20,496 (77.7)	9812 (76.5)	30,308 (77.3)	
Ever	5888 (22.3)	3011 (23.5)	8899 (22.7)	
Physical activity				<0.001
Low	7856 (29.8)	4832 (37.7)	12,688 (32.4)	
Moderate	10,409 (39.5)	4385 (34.2)	14,794 (37.7)	
High	8119 (30.8)	3606 (28.1)	11,725 (29.9)	
High fat diet	5461 (20.7)	2012 (15.7)	7473 (19.1)	<0.001
More vegetables and fruits intake	11,748 (44.5)	4622 (36.0)	16,370 (41.8)	<0.001
Family history of hypertension	4055 (15.4)	3532 (27.5)	7587 (80.6)	<0.001
Type 2 diabetes	1808 (6.9)	1892 (14.8)	3700 (9.5)	<0.001

a For continuous variables, numbers represent the mean ± standard deviation and for categorical variables, numbers represent count (percentage).

Table 2 presents the average concentration levels of three air pollutants. Mean (SD) concentrations were 73.4 (2.6) μg/m^3^ for PM_2.5_, 132.5 (5.8) μg/m^3^ for PM_10_ and 39.9 (3.6) μg/m^3^ for NO_2_. The annual mean levels of exposure to PM_2.5_ and PM_10_ for the all participants far exceeded the standards of World Health Organization air quality guidelines and the Class two limit values of Chinese ambient air quality guideline. We observed high correlations between three air pollutants (Table S1).

**Table 2:** Three-year average exposure levels for the all participants.

Pollutants	Mean ± SD	Median	Min	Max	IQR
PM_2.5_ (μg/m^3^)	73.4 ± 2.6	73.3	68.0	84.9	4.5
PM_10_ (μg/m^3^)	132.5 ± 5.8	133.1	122.4	148.8	10.9
NO_2_ (μg/m^3^)	39.9 ± 3.6	40.3	31.0	49.8	6.4

Abbreviations: PM_2.5_, particulate matter with aerodynamic diameter equal to or less than 2.5  μm; PM_10_, particulate matter with aerodynamic diameter equal to or less than10μm; NO_2_, nitrogen dioxide; SD, standard deviation; Min, minimum; Max, maximum; IQR, interquartile range.

### 3.2 Associations of air pollution with hypertension and blood pressure

We observed positive associations of hypertension with all three pollutants (Fig. 2). In crude models, each 1 μg/m^3^ increase in PM_2.5_, PM_10_ and NO_2_ were associated with an 10.2% (OR: 1.102, 95%CI: 1.093,1.111), 4.8% (OR: 1.048, 95%CI: 1.044, 1.052) and 6.8% (OR: 1.068, 95%CI: 1.062, 1.075) increased odds of hypertension prevalence, respectively. After fully adjusting for potential confounders, each 1 μg/m^3^ increase in PM_2.5_, PM_10_ and NO_2_ was associated with 2.9% (OR: 1.029, 95% CI: 1.001,1.057), 1.5% (OR: 1.015, 95% CI: 1.033, 1.042) and 6.9% (OR: 1.069, 95% CI:1.038, 1.100) higher odds of hypertension prevalence, respectively.

Table 3 summarizes the associations between air pollutants and four blood pressure measurements. In crude models, all three air pollutants were positively associated with systolic blood pressure, diastolic blood pressure, mean arterial pressure and pulse pressure. However, after adjusting for potential confounders, the association between PM_10_ and systolic blood pressure was not observed and all three air pollutants were negatively associated with pulse pressure. The associations of exposure to air pollutants with blood pressure components appeared to be stronger for NO_2_. For different blood pressure measurements, the effect of three air pollutants on diastolic blood pressure was greater than that on systolic blood pressure. In adjusted models, we found that SBP increased by 0.190 mmHg (95% CI: 0.001,0.382) and 0.409 mmHg (95% CI: 0.199, 0.614) associated with per 1 μg/m^3^ increment in PM_2.5_ and NO_2_, respectively, while each 1 μg/m^3^ increment in PM_2.5_, PM_10_ and NO_2_ was associated with 0.342 mmHg (95% CI: 0.228, 0.457), 0.249 mmHg (95% CI: 0.188, 0.311) and 0.797 mmHg (95% CI: 0.667, 0.920) increase in DBP, respectively.

**Table 3:** Adjusted associations of long-term exposure to air pollutants with hypertension and blood pressure, presented per 1 μg/m^3^ of exposure concentration.

Pollutants (μg/m^3^)	Hypertension, OR (95%CI) a	Changes in blood pressure, mmHg (95%CI) a			
		SBP	DBP	MAP	PP
PM_2.5_	1.029 (1.001, 1.057)	0.342 (0.228, 0.457)	0.342 (0.228, 0.457)	0.291 (0.159, 0.425)	−0.149 (-0.240,0.001)
PM_10_	1.015 (1.001, 1.029)	0.249 (0.188, 0.311)	0.249 (0.188, 0.311)	0.182 (0.112, 0.253)	−0.201 (−0.266, −0.134)
NO_2_	1.069 (1.038, 1.100)	0.797 (0.667, 0.920)	0.797 (0.667, 0.920)	0.667 (0.519, 0.810)	−0.378 (−0.509, −0.242)

Abbreviations: PM_2.5_, particle matter with aerodynamic diameter ≤ 2.5 μm; PM_10_, particle matter with aerodynamic diameter ≤ 10 μm; NO_2_, nitrogen dioxide; OR, odds ratio; CI, confidence interval; SBP, systolic blood pressure; DBP, diastolic blood pressure; MAP, mean arterial pressure; PP, pulse pressure.

a Adjusted for sex, age, marital status, education level, income, smoking, alcohol drinking, physical activity, high fat diet, vegetables and fruits intake, family history of hypertension, body mass index, type 2 diabetes.

### 3.3 Stratified analysis for air pollution and hypertension and blood pressure

**Fig. 1:**
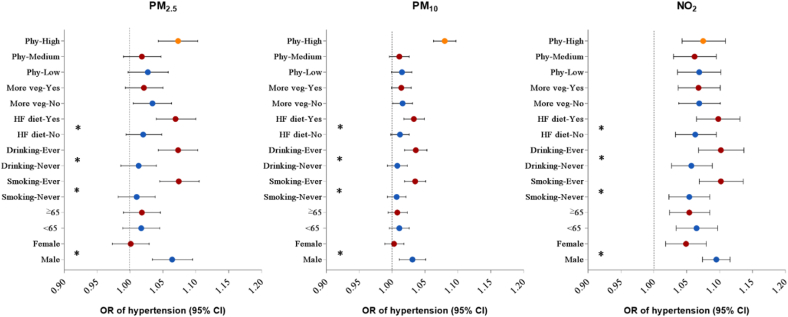
Associations between 1 μg/m^3^ increase in air pollutants and odds ratio (95%CIs) of hypertension, stratified by potential modifiers. Abbreviations: HF diet, high fat diet; More veg: More vegetables and fruits intake; Phy: levels of physical activity. *P for interaction <0.05.

**Fig. 2:**
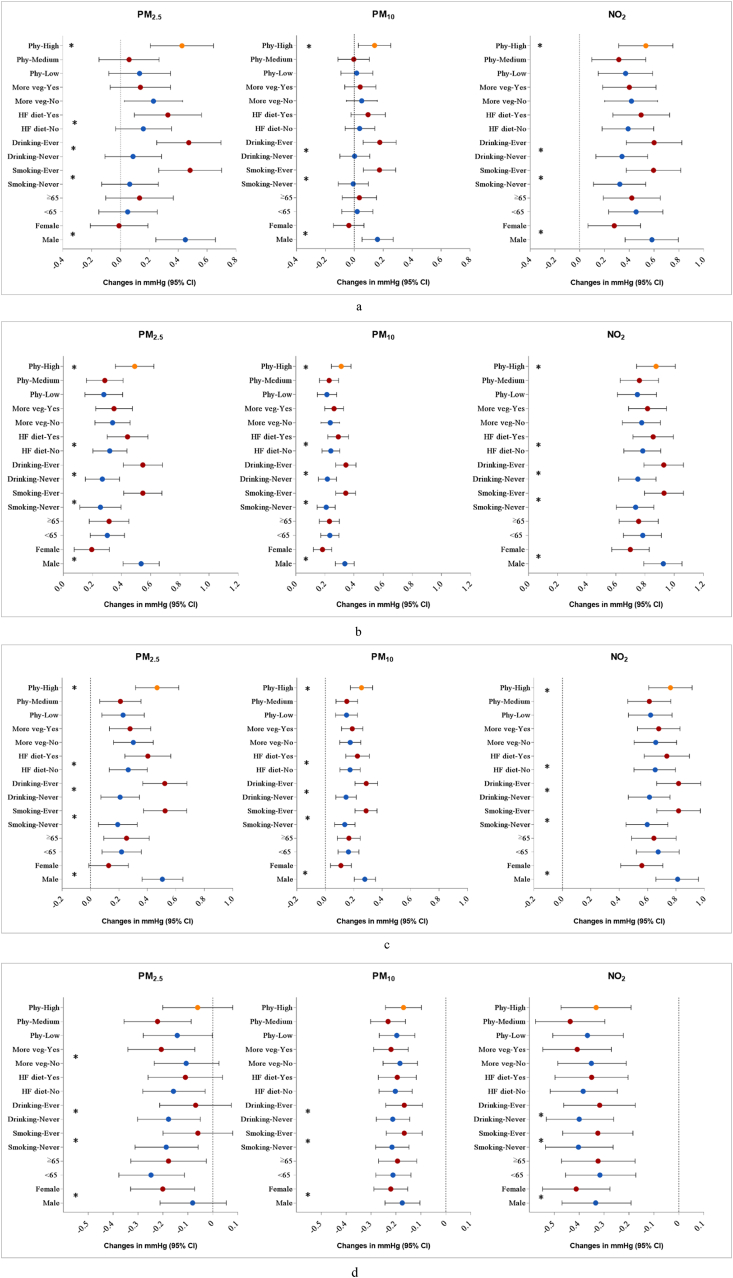
Associations between 1 μg/m^3^ increase in air pollutants and changes (95% CIs) in blood pressure measurements, stratified by potential modifiers. Abbreviations: HF diet, high fat diet; More veg: More vegetables and fruits intake; Phy: levels of physical activity. *P for interaction <0.05.

In stratification analyses by potential modifiers, we observed stronger associations between air pollution exposure and hypertension in males, ever-smokers, ever-drinkers, and those with a high fat diet (Fig. 1). Similar trends were found for SBP, DBP, MAP and PP as well (Fig. 2). Besides, physical activity was also found to be an effect modifier in the associations of air pollution exposure with SBP, DBP and MAP, with greater effect estimates in those participants who had high-level physical activities.

### 3.4 Sensitivity analysis

The sensitivity analyses showed that exclusion of the participants taking anti-hypertensive medicine did not change the results substantially. The results also remained robust after excluding the participants with obesity or T2DM (compare Table S5 with Table 3).

## 4 Discussion

In this large rural population-based study in Central China, we observed positive associations of long-term exposure to air pollution with hypertension prevalence and four blood pressure measurements. Additionally, we found stronger effects of exposure to PM_2.5_ and NO_2_ on blood pressure than PM_10_ exposure. Male participants, participants who ever smoked, participants who ever drunk and participants with a high-fat diet appeared to be more vulnerable to the adverse effects of air pollutants than female participants, never smokers, never drinkers and participants without a high-fat diet.

Previous epidemiology studies have assessed the adverse effect of long-term exposure to air pollution on hypertensive risk (Giorgini et al., 2016; Liang et al., 2014; Yang et al., 2018a). For example, Yang and colleagues included 20, 16 and 19 studies published before May 2017 in their meta analyses to investigate global associations of hypertension with long-term exposure to PM_2.5_, PM_10_ and NO_2_, respectively. They reported that the risk of hypertension increased by 5% per 10 μg/m^3^ increase in PM_2.5_, while no significant associations was reported for the PM_10_ and NO_2_. This study also showed that each 10 μg/m^3^ increment in PM_2.5_, PM_10_ and NO_2_ was associated with higher DBP, with increment ranging from 0.47 to 0.86 mmHg. Apart from reviews above and studies included in reviews, several studies published recently also supported positive associations between long-term exposure to ambient air pollution and hypertension risk and elevated blood pressure. In a study of 3.9 million reproductive-age Chinese adults, an OR of 1.01 (95%CI: 1.007, 1.012) for hypertension was related to a 10 μg/m^3^ increase in PM_2.5_ above an threshold concentration of 47.9 μg/m^3^ (Xie et al., 2018). A study of 15,477 Chinese adults showed that a 10 μg/m^3^ increase in three-year exposure to PM_2.5_, PM_10_ and NO_2_ was significantly associated with ORs of 1.07 (95%CI: 1.02, 1.13), 1.09 (95%CI:1.05,1.12) and 1.19 (95%CI:1.10,1.29) for hypertension, respectively (Yang et al., 2019b). Besides, the strongest associations with ORs of hypertension and increases in blood pressure appeared to be for exposure to NO_2_ in this study. This result is broadly consistent with several studies in which the adverse effect of NO_2_ exposure on health was found to be stronger than the effect of particulate matter exposure (Cesaroni et al., 2013; Hart et al., 2011; Hystad et al., 2013; Jerrett et al., 2013). This higher estimate may be attributed to more accurate exposure assessment, exposure assessment that captured both NO_2_ and PM_2.5_ influences because of the high correlation between air pollutants (Hystad et al., 2013), or better performance of NO2 exposure assessment models in identifying variability in exposure. Overall, our results were generally accordant with these studies and provided additional evidence in support of the adverse effects of air pollution on hypertension.

The findings that exposure to three air pollutants was associated with increased SBP (not PM_10_), DBP, and MAP, and decreased PP is consistent with some previous studies but not with all (Auchincloss et al., 2008; Chan et al., 2015; Honda et al., 2018; Zhang et al., 2018). Chan et al. reported a 10 μg/m^3^ increase in PM_2.5_ to be associated with increased SBP(1.4 mmHg), MAP (0.8 mmHg) and PP(1.0 mmHg), but not DBP(Chan et al., 2015). Among 4121 older Americans (older than 57 years), Honda et al. reported that each 3.91 μg/m^3^ increase in one-year exposure concentration of PM_2.5_ was associated with increased SBP (0.93 mmHg, 95% CI: 0.05, 1.80) and PP (0.89 mmHg, 95% CI: 0.21, 1.58), whereas no associations were observed for DBP and MAP (Honda et al., 2018). Compared with studies mentioned above, the magnitude of blood pressure changed in the present study is slightly larger. The inconsistency among these studies may be due to different study settings, the heterogeneity of the study populations, research methods and adjusted covariates. In addition, we observed that exposure to PM_10_ and NO_2_ was significantly associated with decreased PP. This finding is accordant with an animal toxicologic study reporting that concentrated ambient air particles exposure increased SBP, DBP, MAP, and decreased PP in conscious canines (Bartoli et al., 2009). PP was calculated as the difference of SBP and DBP, and a larger magnitude of elevation was observed for DBP than for SBP in our study, which may ultimately relate to decreased PP. Furthermore, Bartoli et al. pointed out that the decreased pulse pressure induced by concentrated ambient particles exposure in the context of preferential increases in DBP may be accounted by increased peripheral vascular resistance—one of the physiologic mechanisms of blood pressure regulation (Bartoli et al., 2009).

Evidence is inconsistent for sex difference in health effects of air pollution exposure. In the current study, stronger associations of long-term exposure to three air pollutants with hypertension and three blood pressure parameters (SBP, DBP and MAP) were observed among males, which is consistent with a previous study (Dong et al., 2013). Such discrepancy may be related to sex-related differences in the localization of air pollutants during deposition in the airways, the deposition rate decided by the size of body, the size of respiratory tract, and ventilatory parameters (Sacks et al., 2011). Specifically, it has been reported that airways are smaller and airway reactivity is slightly greater for females (Yunginger et al., 1992). Besides, males tend to get involved in more frequent and intense outdoor activities than females do, which may bring about greater pulmonary exposure to ambient air pollution (Abbey et al., 1998; Becklake and Kauffmann, 1999).

The stratified analyses also showed stronger effects of air pollution exposure on hypertension and blood pressure for ever-smokers than for never-smokers, and for ever-drinkers than for never-drinkers. Stronger associations between air pollution exposure and cardiovascular health outcomes in smokers have been observed in other epidemiologic studies as well. Pope et al. found that increases in risks of cardiovascular disease mortality associated with PM_2.5_ elevation were larger for smokers relative to nonsmokers in a prospective cohort study (Pope et al., 2004). Besides, a study carried out among adolescent girls reported that the combined action of cigarette smoking and air pollution exposure (total suspended particulate matter, sulfur dioxide and nitric dioxide) had more detrimental effect on some respiratory and cardiovascular functions than each alone (Turnovska et al., 2007). We consider that these findings are related to the potential biological mechanism of the air pollution-related health effects involving inflammation and oxidative stress. Smoking and drinking can promote responses and even trigger anatomical damage, which may exert additional harmful effect and exacerbate the adverse effects of ambient air pollution as well (Bazzano et al., 2003; O'Keefe et al., 2008; Piano, 2017). However, Pope et al. reported a larger risk of mortality for never smokers in a more recent research (Pope et al., 2019), which is in line with the results of several studies reporting smaller effects of air pollution exposure on blood pressure in smokers (Fuks et al., 2014; Kunzli et al., 2005; Zhang et al., 2018). A possible explanation for this result is that, as some researches indicated, the magnitude of the damaging health effects caused by smoking is relatively higher than that due to PM air pollution exposure (Englert, 2004). Another possible explanation is that smoking and air pollutants may mediate cardiovascular effects through oxidative stress and inflammation by the same pathway and exposure to air pollution might not exert additional effects along the same pathway for the dominant role of smoking in smokers (Zhang et al., 2018). The evidence on modifying effect of smoking on health effect of air pollution have been inconclusive, and well-designed researches are needed to explore the modifying role of such factors.

We also observed greater effects for those participants who had a high-fat diet than for those who did not. One plausible explanation for the result might be that high-fat diet was a key factor related with obesity (Schrauwen and Westerterp, 2000), which was regarded as one of chronic inflammatory conditions that may modulate health effects of air pollution (Sacks et al., 2011). Considerable research showed that obese or overweight individuals are more susceptible to the adverse health effects of particulate matter pollution relative to people of normal weight (Dubowsky et al., 2006; Miller et al., 2007; Zeka et al., 2006). The greater response observed in obese individuals could be attributed to higher dose rates of air pollutants in obese individuals, because individuals who are overweight or obese had an increased in tidal volume and resting minute ventilation, which had been demonstrated in children (Bennett and Zeman, 2004). Overall, the findings from stratified analyses suggest that some unhealthy behavior may aggravate the adverse effects of long-term air pollution exposure on blood pressure.

Additionally, our results indicated a modifying role for physical activity in the effects of air pollution exposure on blood pressure. The high level of physical activity was associated with stronger relationships between air pollutants and SBP, DBP and MAP, compared with low levels. Similar findings were reported in our previously published research (Li et al., 2019), and researches evaluating the effects of air pollution on respiratory outcomes (Strak et al., 2010) and other cardiovascular outcomes (Lin et al., 2017a). Individuals would be exposed to higher concentration of ambient air pollutants due to increased breathing rates and intensity during the outdoor physical activities (Weichenthal et al., 2014). Physically active ones thus suffered from an amplified adverse effect, induced by an increased inhaled quantity and deposition of air pollutants in the body (Strak et al., 2010). With such findings comes concerning about the balance between health gains from physical activity and potential health risks from increased air pollution exposures (Lu et al., 2015).

We acknowledged that our study was subject to several limitations. The primary limitation of this study was its cross-sectional design. We cannot obtain the onset date of hypertension cases, and blood pressure were measured at a single timepoint. Thus, temporal trend of the association and the causal relationships could not be examined. The longitudinal analysis with incidence of hypertension and repeated measurements of blood pressure are needed to confirm our results. Second, recall bias may exist and misclassification might have occurred as all the covariate information was collected using questionnaires. Third, we were not able to apply multi-pollutant models in the analyses because of high correlations among PM_2.5_, PM_10_ and NO_2_, which restricted us to evaluate the effects of multiple pollutants simultaneously. Forth, some important risk factors of elevated blood pressure such as intake of salty food and the food frequency (He et al., 2013; Korhonen et al., 1999; Stamler et al., 1989) were not evaluated in this study due to the lack of data. Another limitation is that our study substantially lacks exposure variability because of the insufficiency of observations with low-level exposure to air pollution. The concentrations of air pollutants in five study regions were much higher compared with some less polluted countries in western Europe or even North America, which suggested that the findings in this study may not be directly applicative to populations exposed to low levels of air pollution, but could provide references for some developing countries with server air pollution.

## 5 Conclusion

Our findings from rural Chinese population suggested that long-term exposure to PM_2.5_, PM_10_ and NO_2_ is significantly associated with increased risk of hypertension and elevation in blood pressure. And the associations can be modified by some behavioral factors including smoking, drinking, high-fat diet and high level of physical activity. Given the severe air pollution and epidemic status of hypertension in rural China, the positive associations observed in our study indicated an urgent need for policy makers to develop effective prevention and intervention policies, and that more attention should be paid to vulnerable population in rural areas.

## Declaration of competing interest

The authors declare no conflicts of interest.
