# Supplementary material for: Associations between long-term exposure to air pollution and blood pressure and effect modifications by behavioral factors
Source: Environ Res. 2020 Mar;182:109109. doi: 10.1016/j.envres.2019.109109 (PMC7043011; doi:10.1016/j.envres.2019.109109)
Supplement: Multimedia component 1 [file mmc1.docx]

**Supplementary Material**

**Table of Contents**

1. Figure S1 The location of five survey sites in the Henan Rural Cohort Study
2. Table S1 Spearman correlation coefficient (P-value) of air pollutants
3. Table S2 Basic demographic and socio-economic characteristics of study participants by five survey sites
4. Table S3 Odd ratios of hypertension associated with an increment of 1 µg/m^3^ in in three air pollutants, stratified by potential modifiers
5. Table S4a Changes in systolic blood pressure (mmHg) associated with an increment of 1µg/m^3^ in in three air pollutants, stratified by potential modifiers
6. Table S4b Changes in diastolic blood pressure (mmHg) associated with an increment of 1µg/m^3^ in in three air pollutants, stratified by potential modifiers
7. Table S4c Changes in mean arterial pressure (mmHg) associated with an increment of 1µg/m^3^ in in three air pollutants, stratified by potential modifiers
8. Table S4d Changes in pulse pressure (mmHg) associated with an increment of 1µg/m^3^ in three air pollutants, stratified by potential modifiers
9. Table S5 Association of long-term exposure to PM_2.5_, PM_10_ and NO_2_ with Hypertension and Blood pressure in sensitivity analyses


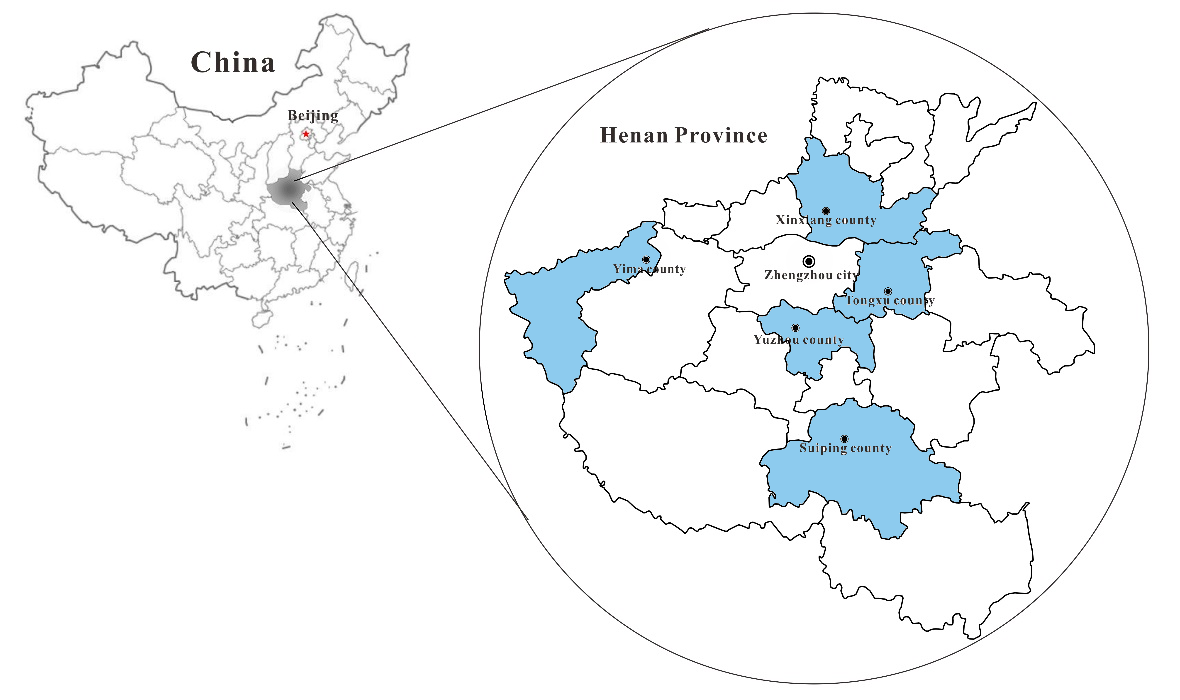


**Figure S1** The locations of five survey sites in the Henan Rural Cohort Study *

* Li N, Chen G, et al. 2019. Associations of long-term exposure to ambient PM_1_ with hypertension and blood pressure in rural Chinese population: The Henan rural cohort study. Environ Int. 128:95-102. https://doi.org/10.1016/j.envint.2019.04.037

**Table S1** Spearman correlation coefficient (*P*-value) of air pollutants

|  | PM_2.5_ | PM_10_ | NO_2_ |
| --- | --- | --- | --- |
| PM_2.5_ | 1.000 | 0.948 (<0.01) | 0.897 (<0.01) |
| PM_10_ |  | 1.000 | 0.964 (<0.01) |
| NO_2_ |  |  | 1.000 |

**Table S2** Basic demographic and socio-economic characteristics of study participants by five survey sites

| Characteristics | **Yuzhou** | |  | **Suiping** | |  | **Tongxu** | |  | **Xinxiang** | |  | **Yima** | |  |
| --- | --- | --- | --- | --- | --- | --- | --- | --- | --- | --- | --- | --- | --- | --- | --- |
|  | Non-HTN (n=6,019) | HTN (n=3,206) | *P* | Non-HTN (n=12,373) | HTN (n=3,656) | *P* | Non-HTN (n=1,353) | HTN (n=1,161) | *P* | Non-HTN (n=6,052) | HTN (n=4,428) | *P* | Non-HTN (n=587) | HTN (n=372) | *P* |
| **Age**, years | 54.0±11.9 | 60.0±9.89 | <0.001 | 54.7±11.7 | 61.0±9.55 | <0.001 | 54.7±12.5 | 62.4±9.48 | <0.001 | 49.1±13.6 | 59.6±10.8 | <0.001 | 54.7±11.4 | 60.2±9.17 | <0.001 |
| **3-year average PM_2.5_**, µg/m^3^ | 76.9±0.92 | 76.9±0.92 |  | 70.9±0.87 | 70.9±0.86 |  | 74.9±1.19 | 74.8±1.28 |  | 74.1±0.84 | 74.1±0.82 |  | 71.6±0.16 | 71.6±0.09 |  |
| **3-year average PM_10_,** µg/m^3^ | 140±1.02 | 140±1.02 |  | 126.3±1.85 | 126.3±1.83 |  | 133±2.26 | 132.9±2.37 |  | 134.5±1.72 | 134.5±1.67 |  | 139.1±0.53 | 139.1±0.93 |  |
| **3-year average NO**_2_, µg/m^3^ | 45±0.50 | 45±0.49 |  | 36.3±0.95 | 36.3±0.95 |  | 37.5±1.17 | 37.5±1.23 |  | 41.1±0.72 | 41.2±0.72 |  | 42.7±0.22 | 42.7±0.02 |  |
| **SBP**, mmHg | 116±11.8 | 147±16.7 | <0.001 | 114±12.0 | 144±16.8 | <0.001 | 120±11.3 | 153±16.5 | <0.001 | 118±11.4 | 147±16.7 | <0.001 | 119±11.1 | 147± 15.0 | <0.001 |
| **DBP**, mmHg | 73.2±7.89 | 88.6±10.5 | <0.001 | 70.7±8.19 | 85.6±10.8 | <0.001 | 74.8±7.24 | 89.5±10.5 | <0.001 | 75.1±7.56 | 89.7±10.3 | <0.001 | 74.9±7.61 | 88.8±9.87 | <0.001 |
| **MAP**, mmHg | 87.6±8.39 | 108±11.0 | <0.001 | 85.1±8.66 | 105±11.2 | <0.001 | 89.8±7.75 | 111±10.7 | <0.001 | 89.4±8.16 | 109±10.9 | <0.001 | 89.5±8.06 | 108±9.95 | <0.001 |
| **PP**, mmHg | 43.2±8.82 | 58.0±14.4 | <0.001 | 43.2±8.84 | 58.4±14.4 | <0.001 | 45.2±8.95 | 64.0±14.9 | <0.001 | 42.9±8.14 | 57.7±14.3 | <0.001 | 43.9±8.17 | 58.6±13.6 | <0.001 |
| **BMI**，kg/m^2^ | 24.6±3.34 | 26.1±3.71 | <0.001 | 23.8±3.26 | 25.3±3.46 | <0.001 | 24.7±3.35 | 26.2±3.75 | <0.001 | 24.7±3.48 | 26.5±3.65 | <0.001 | 24.9±3.20 | 25.9±3.51 | <0.001 |
| **Sex** | | | | | | | | | | | | | | |  |
| Male | 2062 (34.3) | 1180 (36.8) | 0.015 | 5256 (42.5) | 1390 (38.0) | <0.001 | 538 (39.8) | 478 (41.2) | 0.473 | 2297 (38.0) | 1963 (44.3) | <0.001 | 195 (33.2) | 111 (29.8) | 0.274 |
| Female | 3957 (65.7) | 2026 (63.2) |  | 7117 (57.5) | 2266 (62.0) |  | 815 (60.2) | 683 (58.8) |  | 3755 (62.0) | 2465 (55.7) |  | 392 (66.8) | 261 (70.2) |  |
| **Educational level** | | | | | | | | | | | | | | |  |
| Low | 2512 (41.7) | 1764 (55.0) | <0.001 | 5809 (46.9) | 2082 (56.9) | <0.001 | 686 (50.7) | 756 (65.1) | <0.001 | 1520 (25.1) | 2053 (46.4) | <0.001 | 195 (33.2) | 171 (46.0) | <0.001 |
| Medium | 2790 (46.4) | 1168 (36.4) |  | 5156 (41.7) | 1244 (34.0) |  | 482 (35.6) | 285 (24.5) |  | 2499 (41.3) | 1562 (35.3) |  | 283 (48.2) | 154 (41.4) |  |
| High | 717 (11.9) | 274 (8.5) |  | 1408 (11.4) | 330 (9.0) |  | 185 (13.7) | 120 (10.3) |  | 2033 (33.6) | 813 (18.4) |  | 109 (18.6) | 47 (12.6) |  |
| **Marital status** | | | | | | | | | | | | | | |  |
| Married/ cohabiting | 5429 (90.2) | 2736 (85.3) | <0.001 | 11233 (90.8) | 3165 (86.6) | <0.001 | 1245 (92.0) | 1001 (86.2) | <0.001 | 5587 (92.3) | 3946 (89.1) | <0.001 | 529 (90.1) | 325 (87.4) | 0.183 |
| Widowed/single/divorced/separation | 590 (9.8) | 470 (14.7) |  | 1140 (9.2) | 491 (13.4) |  | 108 (8.0) | 160 (13.8) |  | 465 (7.7) | 482 (10.9) |  | 58 (9.9) | 47 (12.6) |  |
| **Individual income per month** | | | | | | | | | | | | | | |  |
| ≤500 RMB | 1948 (32.4) | 1215 (37.9) | <0.001 | 4565 (36.9) | 1509 (41.3) | <0.001 | 429 (31.7) | 441 (38.0) | <0.001 | 1784 (29.5) | 1772 (40.0) | <0.001 | 198 (33.7) | 136 (36.6) | 0.632 |
| 500~1000 RMB | 2298 (38.2) | 1155 (36.0) |  | 3695 (29.9) | 1058 (28.9) |  | 402 (29.7) | 365 (31.4) |  | 2078 (34.3) | 1522 (34.4) |  | 194 (33.0) | 121 (32.5) |  |
| ≥1000 RMB | 1773 (29.5) | 836 (26.1) |  | 4113 (33.2) | 1089 (29.8) |  | 522 (38.6) | 355 (30.6) |  | 2190 (36.2) | 1134 (25.6) |  | 195 (33.2) | 115 (30.9) |  |
| **Smoking** | | | | | | | | | | | | | | |  |
| Never | 4566 (75.9) | 2398 (74.8) | 0.259 | 8705 (70.4) | 2743 (75.0) | <0.001 | 969 (71.6) | 836 (72.0) | 0.829 | 4452 (73.6) | 3129 (70.7) | 0.001 | 453 (77.2) | 289 (77.7) | 0.852 |
| Ever | 1453 (24.1) | 808 (25.2) |  | 3668 (29.6) | 913 (25.0) |  | 384 (28.4) | 325 (28.0) |  | 1600 (26.4) | 1299 (29.3) |  | 134 (22.8) | 83 (22.3) |  |
| **Drinking** | | | | | | | | | | | | | | |  |
| Never | 4601 (76.4) | 2438 (76.0) | 0.670 | 9622 (77.8) | 2923 (80.0) | 0.005 | 990 (73.2) | 845 (72.8) | 0.827 | 4779 (79.0) | 3278 (74.0) | <0.001 | 504 (85.9) | 328 (88.2) | 0.303 |
| Ever | 1418 (23.6) | 768 (24.0) |  | 2751 (22.2) | 733 (20.0) |  | 363 (26.8) | 316 (27.2) |  | 1273 (21.0) | 1150 (26.0) |  | 83 (14.1) | 44 (11.8) |  |
| **High fat diet** | | | | | | | | | | | | | | |  |
| No | 4623 (76.8) | 2572 (80.2) | <0.001 | 9451 (76.4) | 3004 (82.2) | <0.001 | 1178 (87.1) | 1039 (89.5) | 0.06 | 5178 (85.6) | 3869 (87.4) | 0.007 | 493 (84.0) | 327 (87.9) | 0.093 |
| Yes | 1396 (23.2) | 634 (19.8) |  | 2922 (23.6) | 652 (17.8) |  | 175 (12.9) | 122 (10.5) |  | 874 (14.4) | 559 (12.6) |  | 94 (16.0) | 45 (12.1) |  |
| **More vegetables and fruits intake** | | | | | | | | | | | | | | |  |
| No | 4629 (76.9) | 2579 (80.4) | <0.001 | 4406 (35.6) | 1349 (36.9) | 0.151 | 1080 (79.8) | 972 (83.7) | 0.012 | 4162 (68.8) | 3076 (69.5) | 0.446 | 358 (61.0) | 224 (60.2) | 0.811 |
| Yes | 1390 (23.1) | 627 (19.6) |  | 7966 (64.4) | 2306 (63.1) |  | 273 (20.2) | 189 (16.3) |  | 1890 (31.2) | 1352 (30.5) |  | 229 (39.0) | 148 (39.8) |  |
| **Physical activity** | | | | | | | | | | | | | | |  |
| Low | 1864 (31.0) | 1266 (39.5) | <0.001 | 3060 (24.7) | 1063 (29.1) | <0.001 | 166 (12.3) | 217 (18.7) | <0.001 | 2594 (42.9) | 2152 (48.6) | <0.001 | 172 (29.3) | 134 (36.0) | 0.093 |
| Moderate | 2528 (42.0) | 1238 (38.6) |  | 5692 (46.0) | 1698 (46.4) |  | 259 (19.1) | 226 (19.5) |  | 1664 (27.5) | 1069 (24.1) |  | 266 (45.3) | 154 (41.4) |  |
| High | 1627 (27.0) | 702 (21.9) |  | 3621 (29.3) | 895 (24.5) |  | 928 (68.6) | 718 (61.8) |  | 1794 (29.6) | 1207 (27.3) |  | 149 (25.4) | 84 (22.6) |  |
| **Family history of hypertension** | | | | | | | | | | | | | | |  |
| No | 4957 (82.4) | 2318 (72.3) | <0.001 | 10589 (85.6) | 2720 (74.4) | <0.001 | 1138 (84.1) | 966 (83.2) | 0.54 | 5184 (85.7) | 3024 (68.3) | <0.001 | 461 (78.5) | 263 (70.7) | 0.006 |
| Yes | 1062 (17.6) | 888 (27.7) |  | 1784 (14.4) | 936 (25.6) |  | 215 (15.9) | 195 (16.8) |  | 868 (14.3) | 1404 (31.7) |  | 126 (21.5) | 109 (29.3) |  |
| **Type 2 diabetes** | | | | | | | | | | | | | | |  |
| No | 5453 (90.6) | 2700 (84.2) | <0.001 | 11734 (94.8) | 3247 (88.8) | <0.001 | 1228 (90.8) | 991 (85.4) | <0.001 | 5582 (92.2) | 3655 (82.5) | <0.001 | 540 (92.0) | 314 (84.4) | <0.001 |
| Yes | 562 (9.3) | 505 (15.8) |  | 632 (5.1) | 404 (11.1) |  | 120 (8.9) | 169 (14.6) |  | 451 (7.5) | 757 (17.1) |  | 43 (7.3) | 57 (15.3) |  |
| **Taking anti-HTN medicine** | - | 1602 (50.0) | - | - | 1863 (51.0) | - | - | 430 (37.0) | - | - | 2240 (50.6) | - | - | 171 (46.0) | - |
| **3-year average temperature (℃)** | 15.8 | |  | 16.2 | |  | 14.3 | |  | 16.2 | |  | 16.2 | |  |

**Table S3** Odd ratios of hypertension associated with an increment of 1 µg/m^3^ in in three air pollutants, stratified by potential modifiers

| Modifiers | PM_2.5_ * | | PM_10_ * | | NO_2_ * | |
| --- | --- | --- | --- | --- | --- | --- |
|  | mmHg (95%CI) | P_interaction_ | mmHg (95%CI) | P_interaction_ | mmHg (95%CI) | P_interaction_ |
| Gender |  |  |  |  |  |  |
| Male | 1.064 (1.034, 1.095) | < 0.001 | 1.031 (1.011, 1.051) | < 0.001 | 1.095 (1.074, 1.116) | < 0.001 |
| Female | 1.001 (0.973, 1.029) |  | 1.003 (0.989, 1.018) |  | 1.049 (1.018, 1.080) |  |
| Age |  |  |  |  |  |  |
| <65 | 1.013 (0.987, 1.039) | 0.317 | 1.011 (0.996, 1.026) | 0.516 | 1.065 (1.034, 1.097) | 0.135 |
| ≥65 | 1.003 (0.974, 1.033) |  | 1.008 (0.994, 1.023) |  | 1.054 (1.024, 1.085) |  |
| Smoking |  |  |  |  |  |  |
| Never | 1.010 (0.982, 1.038) | < 0.001 | 1.007 (0.993, 1.021) | < 0.001 | 1.054 (1.023, 1.085) | < 0.001 |
| Ever | 1.074 (1.045, 1.105) |  | 1.035 (1.019, 1.051) |  | 1.102 (1.069, 1.136) |  |
| Drinking |  |  |  |  |  |  |
| Never | 1.013 (0.986, 1.040) | < 0.001 | 1.008 (0.993, 1.023) | < 0.001 | 1.057 (1.027, 1.089) | < 0.001 |
| Ever | 1.073 (1.043, 1.103) |  | 1.036 (1.019, 1.053) |  | 1.102 (1.068, 1.137) |  |
| More vegetables and fruits intake | |  |  |  |  |  |
| No | 1.034 (1.005, 1.063) | 0.242 | 1.016 (1.001, 1.031) | 0.739 | 1.069 (1.038, 1.101) | 0.930 |
| Yes | 1.021 (0.993, 1.050) |  | 1.014 (0.999, 1.029) |  | 1.068 (1.037, 1.101) |  |
| High-fat diet | |  |  |  |  |  |
| No | 1.020 (0.994, 1.048) | < 0.001 | 1.012 (0.998, 1.026) | < 0.001 | 1.063 (1.033, 1.095) | < 0.001 |
| Yes | 1.069 (1.040, 1.100) |  | 1.033 (1.018, 1.049) |  | 1.098 (1.065, 1.131) |  |
| Physical activity | |  |  |  |  |  |
| Low | 1.027 (0.997, 1.058) | Reference | 1.015 (0.999, 1.030) | Reference | 1.069 (1.036, 1.102) | Reference |
| Moderate | 1.018 (0.990, 1.047) | 0.444 | 1.011 (0.996, 1.026) | 0.459 | 1.062 (1.030, 1.095) | 0.450 |
| High | 1.073 (1.043, 1.103) | 0.223 | 1.080 (1.063, 1.097) | 0.324 | 1.075 (1.043, 1.109) | 0.492 |

**Table S4a** Changes in systolic blood pressure (mmHg) associated with an increment of 1µg/m^3^ in in three air pollutants, stratified by potential modifiers

| Modifiers | PM_2.5_ * | | PM_10_ * | | NO_2_ * | |
| --- | --- | --- | --- | --- | --- | --- |
|  | mmHg (95%CI) | P_interaction_ | mmHg (95%CI) | P_interaction_ | mmHg (95%CI) | P_interaction_ |
| Gender |  |  |  |  |  |  |
| Male | 0.449 (0.245, 0.657) | < 0.001 | 0.160 (0.054, 0.269) | < 0.001 | 0.582 (0.366, 0.795) | < 0.001 |
| Female | -0.010 (-0.209, 0.190) |  | -0.038(-0.144, 0.067) |  | 0.278 (0.066, 0.490) |  |
| Age |  |  |  |  |  |  |
| <65 | 0.050 (-0.150, 0.254) | 0.312 | 0.020 (-0.087, 0.129) | 0.673 | 0.455 (0.233, 0.671) | 0.542 |
| ≥65 | 0.132 (-0.102, 0.366) |  | 0.035 (-0.083, 0.153) |  | 0.420 (0.190, 0.650) |  |
| Smoking |  |  |  |  |  |  |
| Never | 0.064 (-0.130, 0.262) | < 0.001 | -0.008(-0.111, 0.097) | < 0.001 | 0.323 (0.111, 0.531) | < 0.001 |
| Ever | 0.482 (0.265, 0.700) |  | 0.174 (0.062, 0.286) |  | 0.595 (0.375, 0.815) |  |
| Drinking |  |  |  |  |  |  |
| Never | 0.087 (-0.107, 0.284) | < 0.001 | 0.002 (-0.100, 0.107) | < 0.001 | 0.341 (0.130, 0.548) | < 0.001 |
| Ever | 0.473 (0.251, 0.696) |  | 0.176 (0.061, 0.290) |  | 0.600 (0.376, 0.824) |  |
| More vegetables and fruits intake | |  |  |  |  |  |
| No | 0.228 (0.028, 0.430) | 0.235 | 0.051 (-0.054, 0.159) | 0.761 | 0.417 (0.201, 0.628) | 0.749 |
| Yes | 0.138 (-0.071, 0.347) |  | 0.041 (-0.066, 0.149) |  | 0.400 (0.185, 0.615) |  |
| High-fat diet | |  |  |  |  |  |
| No | 0.158 (-0.034, 0.353) | 0.041 | 0.037 (-0.064, 0.141) | 0.115 | 0.390 (0.180, 0.597) | 0.075 |
| Yes | 0.328 (0.096, 0.560) |  | 0.095 (-0.023, 0.214) |  | 0.495 (0.267, 0.724) |  |
| Physical activity | |  |  |  |  |  |
| Low | 0.132 (-0.081, 0.347) | Reference | 0.016 (-0.093, 0.128) | Reference | 0.370 (0.149, 0.588) | Reference |
| Moderate | 0.060 (-0.149, 0.268) | 0.376 | -0.004(-0.113, 0.104) | 0.563 | 0.315 (0.097, 0.532) | 0.343 |
| High | 0.426 (0.207, 0.644) | 0.001 | 0.140 (0.028, 0.252) | 0.002 | 0.533 (0.314, 0.752) | 0.010 |

**Table S4b** Changes in diastolic blood pressure (mmHg) associated with an increment of 1µg/m^3^ in in three air pollutants, stratified by potential modifiers

| Modifiers | PM_2.5_ * | | PM_10_ * | | NO_2_ * | |
| --- | --- | --- | --- | --- | --- | --- |
|  | mmHg (95%CI) | P_interaction_ | mmHg (95%CI) | P_interaction_ | mmHg (95%CI) | P_interaction_ |
| Gender |  |  |  |  |  |  |
| Male | 0.534 (0.410, 0.658) | < 0.001 | 0.337 (0.272, 0.401) | < 0.001 | 0.925 (0.791, 1.053) | < 0.001 |
| Female | 0.195 (0.075, 0.316) |  | 0.184 (0.121, 0.247) |  | 0.699 (0.571, 0.828) |  |
| Age |  |  |  |  |  |  |
| <65 | 0.302 (0.185, 0.420) | 0.805 | 0.234 (0.172, 0.297) | 0.878 | 0.784 (0.652, 0.911) | 0.392 |
| ≥65 | 0.314 (0.178, 0.450) |  | 0.231 (0.162, 0.299) |  | 0.756 (0.622, 0.890) |  |
| Smoking |  |  |  |  |  |  |
| Never | 0.255 (0.114, 0.396) | < 0.001 | 0.209 (0.147, 0.271) | < 0.001 | 0.735 (0.603, 0.860) | < 0.001 |
| Ever | 0.546 (0.414, 0.677) |  | 0.343 (0.275, 0.410) |  | 0.930 (0.796, 1.063) |  |
| Drinking |  |  |  |  |  |  |
| Never | 0.268 (0.151, 0.386) | < 0.001 | 0.217 (0.155, 0.279) | < 0.001 | 0.750 (0.619, 0.875) | < 0.001 |
| Ever | 0.546 (0.412, 0.681) |  | 0.344 (0.275, 0.413) |  | 0.928 (0.792, 1.063) |  |
| More vegetables and fruits intake | |  |  |  |  |  |
| No | 0.338 (0.217, 0.459) | 0.818 | 0.237 (0.173, 0.301) | 0.188 | 0.777 (0.644, 0.905) | 0.222 |
| Yes | 0.348 (0.222, 0.474) |  | 0.263 (0.199, 0.328) |  | 0.817 (0.687, 0.947) |  |
| High-fat diet | |  |  |  |  |  |
| No | 0.319 (0.203, 0.436) | 0.015 | 0.241 (0.180, 0.303) | 0.023 | 0.784 (0.654, 0.908) | 0.046 |
| Yes | 0.441 (0.301, 0.581) |  | 0.292 (0.220, 0.363) |  | 0.855 (0.717, 0.994) |  |
| Physical activity | |  |  |  |  |  |
| Low | 0.278 (0.149, 0.407) | Reference | 0.214 (0.148, 0.281) | Reference | 0.747 (0.610, 0.878) | Reference |
| Moderate | 0.285 (0.159, 0.410) | 0.884 | 0.229 (0.163, 0.294) | 0.508 | 0.761 (0.629, 0.892) | 0.691 |
| High | 0.490 (0.358, 0.622) | <0.001 | 0.312 (0.245, 0.379) | <0.001 | 0.875 (0.742, 1.007) | 0.001 |

**Table S4c** Changes in mean arterial pressure (mmHg) associated with an increment of 1µg/m^3^ in in three air pollutants, stratified by potential modifiers

| Modifiers | PM_2.5_ * | | PM_10_ * | | NO_2_ * | |
| --- | --- | --- | --- | --- | --- | --- |
|  | mmHg (95%CI) | P_interaction_ | mmHg (95%CI) | P_interaction_ | mmHg (95%CI) | P_interaction_ |
| Gender |  |  |  |  |  |  |
| Male | 0.505 (0.363, 0.649) | < 0.001 | 0.278 (0.204, 0.353) | < 0.001 | 0.811 (0.658, 0.958) | < 0.001 |
| Female | 0.127 (-0.012, 0.266) |  | 0.110 (0.037, 0.183) |  | 0.559 (0.411, 0.707) |  |
| Age |  |  |  |  |  |  |
| <65 | 0.218 (0.081, 0.357) | 0.526 | 0.163 (0.090, 0.237) | 0.905 | 0.674 (0.521, 0.822) | 0.433 |
| ≥65 | 0.253 (0.094, 0.413) |  | 0.166 (0.085, 0.246) |  | 0.644 (0.487, 0.801) |  |
| Smoking |  |  |  |  |  |  |
| Never | 0.191 (0.056, 0.328) | < 0.001 | 0.137 (0.065, 0.209) | < 0.001 | 0.597 (0.448, 0.742) | < 0.001 |
| Ever | 0.524 (0.373, 0.676) |  | 0.287 (0.209, 0.364) |  | 0.818 (0.664, 0.971) |  |
| Drinking |  |  |  |  |  |  |
| Never | 0.208 (0.073, 0.344) | < 0.001 | 0.146 (0.074, 0.218) | < 0.001 | 0.613 (0.464, 0.757) | < 0.001 |
| Ever | 0.522 (0.367, 0.677) |  | 0.288 (0.209, 0.368) |  | 0.818 (0.663, 0.974) |  |
| More vegetables and fruits intake | |  |  |  |  |  |
| No | 0.301 (0.162, 0.441) | 0.662 | 0.175 (0.102, 0.250) | 0.539 | 0.657 (0.505, 0.804) | 0.582 |
| Yes | 0.278 (0.133, 0.423) |  | 0.190 (0.115, 0.264) |  | 0.678 (0.528, 0.828) |  |
| High-fat diet | |  |  |  |  |  |
| No | 0.265 (0.132, 0.400) | 0.017 | 0.174 (0.103, 0.245) | 0.039 | 0.653 (0.504, 0.796) | 0.045 |
| Yes | 0.403 (0.242, 0.565) |  | 0.226 (0.144, 0.309) |  | 0.735 (0.576, 0.894) |  |
| Physical activity | |  |  |  |  |  |
| Low | 0.229 (0.080, 0.378) | Reference | 0.149 (0.072, 0.226) | Reference | 0.621 (0.465, 0.772) | Reference |
| Moderate | 0.210 (0.065, 0.354) | 0.734 | 0.151 (0.076, 0.227) | 0.915 | 0.612 (0.460, 0.763) | 0.822 |
| High | 0.468 (0.317, 0.620) | <0.001 | 0.255 (0.177, 0.333) | <0.001 | 0.761 (0.608, 0.913) | 0.002 |

**Table S4d** Changes in pulse pressure (mmHg) associated with an increment of 1µg/m^3^ in three air pollutants, stratified by potential modifiers

| Modifiers | PM_2.5_ * | | PM_10_ * | | NO_2_ * | |
| --- | --- | --- | --- | --- | --- | --- |
|  | mmHg (95%CI) | P_interaction_ | mmHg (95%CI) | P_interaction_ | mmHg (95%CI) | P_interaction_ |
| Gender |  |  |  |  |  |  |
| Male | -0.081 (-0.212, 0.054) | < 0.001 | -0.175(-0.244, -0.104) | < 0.001 | -0.334 (-0.469, -0.192) | < 0.001 |
| Female | -0.201 (-0.330, -0.073) |  | -0.221(-0.289, -0.153) |  | -0.412 (-0.547, -0.276) |  |
| Age |  |  |  |  |  |  |
| <65 | -0.248 (-0.377, -0.114) | 0.181 | -0.212 (-0.281, -0.140) | 0.431 | -0.317 (-0.455, -0.173) | 0.859 |
| ≥65 | -0.178 (-0.329, -0.026) |  | -0.194 (-0.270, -0.117) |  | -0.324 (-0.471, -0.176) |  |
| Smoking |  |  |  |  |  |  |
| Never | -0.187 (-0.312, -0.059) | 0.010 | -0.216 (-0.282, -0.148) | 0.024 | -0.403 (-0.535, -0.264) | 0.028 |
| Ever | -0.060 (-0.200, 0.080) |  | -0.167 (-0.239, -0.095) |  | -0.325 (-0.466, -0.184) |  |
| Drinking |  |  |  |  |  |  |
| Never | -0.178 (-0.302, -0.050) | 0.036 | -0.213 (-0.279, -0.145) | 0.046 | -0.400 (-0.532, -0.262) | 0.028 |
| Ever | -0.069 (-0.213, 0.074) |  | -0.167 (-0.241, -0.094) |  | -0.318 (-0.462, -0.175) |  |
| More vegetables and fruits intake | |  |  |  |  |  |
| No | -0.107 (-0.235, 0.025) | 0.041 | -0.184 (-0.252, -0.114) | 0.090 | -0.351 (-0.486, -0.211) | 0.101 |
| Yes | -0.207 (-0.341, -0.072) |  | -0.220 (-0.290, -0.151) |  | -0.408 (-0.546, -0.271) |  |
| High-fat diet | |  |  |  |  |  |
| No | -0.158 (-0.281, -0.031) | 0.374 | -0.203 (-0.268, -0.135) | 0.752 | -0.384 (-0.516, -0.247) | 0.372 |
| Yes | -0.110 (-0.260, 0.039) |  | -0.195 (-0.271, -0.119) |  | -0.350 (-0.497, -0.204) |  |
| Physical activity | |  |  |  |  |  |
| Low | -0.143 (-0.279, -0.002) | Reference | -0.197 (-0.267, -0.124) | Reference | -0.367 (-0.506, -0.223) | Reference |
| Moderate | -0.222 (-0.356, -0.087) | 0.132 | -0.232 (-0.302, -0.162) | 0.130 | -0.436 (-0.575, -0.297) | 0.066 |
| High | -0.061 (-0.201, 0.080) | 0.157 | -0.170 (-0.242, -0.098) | 0.303 | -0.332 (-0.473, -0.192) | 0.394 |

Abbreviations: PM_2.5_, particle matter with aerodynamic diameter ≤ 2.5μm; PM_10_, particle matter with aerodynamic diameter ≤ 10μm; NO_2_, Nitrogen dioxide; CI, confidence interval.

*Adjusted for sex, age, marital status, education level, income smoking, alcohol drinking, physical activity, high fat diet, vegetables and fruits intake, family history of hypertension, body mass index, type 2 diabetes

**Table S5** Association of long-term exposure to PM_2.5_, PM_10_ and NO_2_ with Hypertension and Blood pressure in sensitivity analyses

**Excluding individuals taking anti-hypertensive medicine**

| Air pollutants | OR of hypertension (95% CI) | Changes in mmHg (95% CI) | | | |
| --- | --- | --- | --- | --- | --- |
|  |  | SBP | DBP | MAP | PP |
| PM_2.5_ | 1.072 (1.039, 1.107) | 0.363 (0.173, 0.555) | 0.438 (0.320, 0.556) | 0.413 (0.279,0.548) | -0.072 (-0.193, 0.053) |
| PM_10_ | 1.034 (1.017, 1.052) | 0.141 (0.040, 0.244) | 0.298 (0.235, 0.360) | 0.246 (0.174,0.317) | -0.155 (-0.220, -0.088) |
| NO_2_ | 1.112 (1.073, 1.152) | 0.562 (0.352, 0.767) | 0.887(0.755, 1.014) | 0.779 (0.629, 0.923) | -0.314 (-0.443, -0.179) |

**Excluding individuals with obesity or T2DM**

| Air pollutants | OR of hypertension (95% CI) | Changes in mmHg (95% CI) | | | |
| --- | --- | --- | --- | --- | --- |
|  |  | SBP | DBP | MAP | PP |
| PM_2.5_ | 1.012 (0.981,1.045) | 0.139 (-0.075, 0.357) | 0.294 (0.164, 0.425) | 0.242 (0.092, 0.393) | -0.150 (-0.285, -0.009) |
| PM_10_ | 1.009 (0.992, 1.026) | 0.036( -0.078, 0.152) | 0.232 (0.163, 0.301) | 0.784 (0.638, 0.923) | -0.194 (-0.266, -0.118) |
| NO_2_ | 1.059 (1.024, 1.095) | 0.417 (0.182, 0.647) | 0.784 (0.638, 0.923) | 0.662 (0.495, 0.822) | -0.355 (-0.500, -0.203) |

**Using fixed-effect model**

| Air pollutants | OR of hypertension (95% CI) | Changes in mmHg (95% CI) | | | |
| --- | --- | --- | --- | --- | --- |
|  |  | SBP | DBP | MAP | PP |
| PM_2.5_ | 1.079 (1.069,1.090) | 0.807 (0.734, 0.880) | 0.592 (0.548, 0.636) | 0.664 (0.613, 0.714) | 0.215 (0.168, 0.261) |
| PM_10_ | 1.037 (1.033, 1.042) | 0.381 (0.348, 0.413) | 0.295 (0.275, 0.314) | 0.323 (0.301, 0.346) | 0.086 (0.065, 0.107) |
| NO_2_ | 1.050 (1.043, 1.057) | 0.488 (0.437, 0.540) | 0.411 (0.380, 0.442) | 0.437 (0.401, 0.472) | 0.078 (0.045, 0.111) |

Abbreviations: PM_2.5_, particle matter with aerodynamic diameter ≤ 2.5μm; PM_2.5_, particle matter with aerodynamic diameter ≤ 2.5μm; OR, odds ratio; CI, confidence interval; SBP, systolic blood pressure; DBP, diastolic blood pressure; MAP, mean arterial pressure; PP, pulse pressure
